# Optimizing pediatric rheumatology training of adult rheumatology fellows

**DOI:** 10.1186/1546-0096-10-S1-A10

**Published:** 2012-07-13

**Authors:** Eliezer Mendelev, Robert P Sundel

**Affiliations:** 1 Children's Hospital, Boston, MA, USA

## Purpose

The approximately 250 board certified Pediatric Rheumatologists in the United States are unable to see all of the children in need of specialized rheumatologic care. Consequently, many pediatric patients receive this care from practitioners trained in internal medicine and/or adult rheumatology. With only 10-20 fellows graduating from pediatric rheumatology training programs each year, reliance on non-pediatricians is likely to continue for the foreseeable future. Little is known about how best to prepare adult-trained providers to care for children. We examined the views of adult rheumatology fellows (ARF) training in a pediatric rheumatology clinic in an attempt to learn how to optimize a pediatric training program for adult rheumatologists.

## Methods

Three to six ARF have received weekly pediatric rheumatology training at our institution annually since 2005. ARF were surveyed at the start and end of their 3-month pediatric rotation in an attempt to gauge their overall comfort level treating pediatric patients. Further survey items focused on identifying specific aspects of pediatric care viewed as most dissimilar from adult medicine, and diagnoses about which the ARF most wanted to learn. Follow-up questionnaires asked for rank ordering of major strengths and weaknesses of the pediatric rheumatology training. Visual analog and ordinal scales were utilized, and respondents were asked to elaborate on their answers using unstructured specification.

## Results

Initially, adult rheumatology fellows reported low comfort levels treating pediatric patients, with a median score of 25 on a 100 mm visual analog scale ranging from “utterly uncomfortable seeing children” to “completely comfortable with my ability to assess children.” Upon completion of the 3-month long pediatric rotation, comfort levels rose to a median of 75 on the 100 mm scale. The value of the pediatric rheumatology experience was given a median ranking of 93 on a 100 mm VAS ranging from “complete waste of time” to “outstanding experience.” The aspect of care that was reported to be most different between pediatric and adult medicine before the rotation was “growth and development issues,” with all currently processed responses ranking it as such. The diagnoses unique to children about which fellows most wanted to learn were, in order, juvenile arthritis, SLE, and Kawasaki disease. Responses were positively correlated with the incidence of the condition.

## Conclusion

This is a preliminary report of an ongoing prospective analysis of adult rheumatology fellows’ training in pediatric rheumatology. Results support an apparent efficacy of rotations in pediatric rheumatology to prepare ARF for the care of pediatric patients. Data concerning particular areas of interest and perceived need are being identified. Analysis remains incomplete and numbers analyzed to date are small. Ongoing data analysis, as well as comparison of subjective responses with objective measures of performance, may identify specific approaches that could optimize training of ARF.

## Disclosure

Eliezer Mendelev: None; Robert P. Sundel: None.

